# Timing and Clinical Impact of Opportunistic Infections in Pediatric Kidney Transplant Recipients: A 10-Year Single-Center Experience

**DOI:** 10.3390/jcm15124668

**Published:** 2026-06-16

**Authors:** Antonia Kondou, John Dotis, Vasiliki Karava, Eleni Papadimitriou, Charalampos Agakidis, Grigorios Myserlis, Lemonia Skoura, Dimitrios Zafeiriou, Georgios Tsoulfas, Georgia Gioula, Georgios Papazisis, Nikoleta Printza

**Affiliations:** 1 First Department of Pediatrics, Aristotle University of Thessaloniki, Hippokration Hospital, 54642 Thessaloniki, Greece; antkontou@auth.gr (A.K.); epapadimitriu@gmail.com (E.P.); cagakidis@auth.gr (C.A.); dizafeir@auth.gr (D.Z.); nprintza@gmail.com (N.P.); 2 Third Department of Pediatrics, Aristotle University of Thessaloniki, Hippokration Hospital, 54642 Thessaloniki, Greece; 3 Pediatric Nephrology Unit, “Aghia Sophia” Children’s Hospital, 11527 Athens, Greece; vasilikikarava@hotmail.fr; 4 Department of Transplantation Surgery, Aristotle University of Thessaloniki, Hippokration Hospital, 54642 Thessaloniki, Greece; grammateia@ippokratio.gr (G.M.); tsoulfasg@auth.gr (G.T.); 5 Department of Microbiology, AHEPA University General Hospital, 54636 Thessaloniki, Greece; lemskour@auth.gr; 6 Department of Microbiology, School of Medicine, Aristotle University of Thessaloniki, 54636 Thessaloniki, Greece; ggioula@auth.gr; 7 Laboratory of Clinical Pharmacology, School of Medicine, Aristotle University of Thessaloniki, 54636 Thessaloniki, Greece; papazisg@auth.gr

**Keywords:** pediatric kidney transplantation, opportunistic infections, BK virus, cytomegalovirus, fungal infections, graft dysfunction, immunosuppression

## Abstract

**Background/Objectives**: Opportunistic infections remain clinically important after kidney transplantation and may contribute to morbidity and graft dysfunction in pediatric recipients. Data regarding their timing, spectrum and clinical course in children remain limited. **Methods**: We retrospectively reviewed pediatric kidney transplant recipients followed at a single tertiary center between 2014 and 2024. Demographic and clinical characteristics, infection type, timing after transplantation, management and outcomes were recorded. Infection incidence was assessed at the patient level, whereas pathogen distribution and timing were analyzed per infection episode. **Results**: Twenty-seven pediatric kidney transplant recipients were included, with a mean follow-up of 5.6 years. Ten patients (37.0%) developed at least one clinically significant opportunistic infection, and one patient experienced two distinct episodes, resulting in 11 infection events. BK virus was the most frequent pathogen, followed by fungal infections and cytomegalovirus (CMV). Five episodes (45.5%) occurred within the first post-transplant year, whereas six (54.5%) occurred later during follow-up. Late infections included CMV, fungal infections, BK virus and West Nile virus. Most infections resolved after targeted management without persistent graft impairment; however, one patient developed biopsy-confirmed BK virus-associated nephropathy with sustained graft dysfunction. No infection-related mortality was observed. **Conclusions**: Clinically significant opportunistic infections occurred both early and late after pediatric kidney transplantation, with more than half of all infectious episodes developing beyond the first post-transplant year. Although overall outcomes were favorable, BK virus-associated nephropathy remained clinically relevant because of its impact on graft function.

## 1. Introduction

Kidney transplantation remains the preferred treatment modality for children with end-stage kidney disease (ESKD), providing better long-term survival and improved overall quality of life compared with dialysis [1]. Over the past decades, improvements in surgical approaches, immunosuppressive strategies and post-transplant monitoring have contributed substantially to better patient and graft outcomes [2].

Infectious complications continue to represent a major source of morbidity among pediatric kidney transplant recipients [3]. Chronic immunosuppressive therapy predisposes pediatric transplant recipients to a broad spectrum of viral, fungal and other opportunistic infections that may necessitate prolonged treatment, hospitalization, adjustment of immunosuppressive regimens, or evaluation for possible graft dysfunction [3]. Preventive strategies, including pre-transplant immunization and post-transplant surveillance protocols, remain essential components of transplant care [4]. Opportunistic infections may present non-specific or atypical clinical manifestations in immunocompromised hosts, making prompt diagnosis more difficult [5,6]. Their clinical impact depends not only on the causative pathogen, but also on the intensity of immunosuppression, the interval since transplantation, and the recipient’s previous exposure to common viral agents.

The epidemiology of post-transplant infections generally follows a characteristic temporal distribution and is often categorized into early (0–1 month), intermediate (1–6 months), and late (>6 months) post-transplant periods [3]. Opportunistic infections are more frequently encountered during periods of intensified immunosuppression, particularly within the first months after transplantation [3,7]. However, clinically significant infections may also occur during long-term maintenance immunosuppression and may reflect cumulative immunosuppressive exposure, individual susceptibility, or changes in local epidemiology [8,9]. Although most surveillance protocols primarily emphasize the early post-transplant period, late infectious complications may still contribute significantly to morbidity and graft dysfunction during extended follow-up. In the present study, a 12-month cutoff was used to distinguish infections occurring during the first post-transplant year from those developing later during follow-up.

In children, infection risk may be further influenced by limited pre-transplant exposure to common viral pathogens, thereby increasing the likelihood of primary infection after transplantation [6,7]. This vulnerability may persist despite advances in prophylactic strategies and virological surveillance. Pediatric data regarding the timing, clinical presentation, management, and outcomes of clinically significant opportunistic infections remain relatively limited, particularly in cohorts with extended follow-up [7].

Most published pediatric studies have focused primarily on specific viral pathogens, such as CMV, EBV, or BK virus, whereas data addressing the broader spectrum of clinically significant opportunistic infections remain scarce [3,4,5,6]. Furthermore, information regarding the timing and clinical impact of these infections during long-term follow-up is limited.

The present study evaluated the incidence, timing, pathogen spectrum, management and clinical outcomes of clinically significant opportunistic infections in pediatric kidney transplant recipients followed over a 10-year period at a tertiary referral center.

## 2. Materials and Methods

### 2.1. Study Design and Population

A retrospective observational cohort study was conducted in pediatric patients who underwent kidney transplantation and were followed at the Transplantation Clinic of a tertiary referral center between January 2014 and December 2024. All consecutive pediatric kidney transplant recipients meeting these criteria during the study period were included in the analysis. In total, 27 pediatric kidney transplant recipients were analyzed. Follow-up extended from transplantation to the most recent available clinical assessment or graft loss, when applicable.

### 2.2. Data Collection and Clinical Variables

Clinical data were retrieved from patient medical records, including electronic databases and archived files. Recorded variables included age at transplantation, sex, underlying kidney disease, donor type, immunosuppressive therapy and duration of follow-up. Additional collected variables included infection timing after transplantation, hospitalization requirements, laboratory findings, graft-function changes and therapeutic interventions related to infectious episodes.

Information on opportunistic infections included pathogen type, timing of onset after transplantation, diagnostic method, management and clinical course. Because asymptomatic viral replication is relatively common after transplantation, only clinically significant infectious episodes were included in the analysis.

In patients with more than one infection, each episode was recorded separately. Management data included changes in immunosuppressive therapy, antimicrobial, antiviral, or antifungal treatment and use of adjunctive therapies. Clinical response and infection-related outcomes were recorded, including resolution, persistence or progression of infection and impact on graft function. When applicable, infection-related graft dysfunction was evaluated in relation to each patient’s baseline renal function and overall clinical course.

### 2.3. Immunosuppressive Regimen and Infection Prophylaxis

All patients received triple immunosuppressive therapy consisting of a calcineurin inhibitor, an antiproliferative agent, and corticosteroids, according to standard clinical practice. The maintenance regimen was based predominantly on tacrolimus, mycophenolate mofetil, and corticosteroids. Immunosuppressive therapy was individualized according to immunological risk profile, graft function, and rejection-related clinical events. In cases of biopsy-proven or clinically suspected rejection, immunosuppression was intensified according to established international treatment protocols and subsequently adjusted based on clinical response and graft function.

All patients received antimicrobial prophylaxis with trimethoprim-sulfamethoxazole after transplantation, administered three times weekly for at least 6 months, unless contraindicated [10]. Antiviral prophylaxis with oral valganciclovir was administered to all patients except donor-negative/recipient-negative cytomegalovirus (CMV) pairs. Prophylaxis consisted of an induction phase followed by maintenance therapy for at least 6 months and usually for 12 months, as recommended in pediatric kidney transplant recipients [11].

Routine virological surveillance for CMV, Epstein–Barr virus (EBV), and BK virus (BKV) was performed monthly during the first 6 months after transplantation and every 3 months thereafter, or earlier when clinically indicated [9,11,12,13]. Additional virological assessment was performed in the presence of fever, unexplained graft dysfunction, cytopenias, or other clinical findings suggestive of opportunistic infection.

### 2.4. Definitions and Diagnostic Criteria

Opportunistic infections were defined as infections occurring in the setting of immunosuppression and caused by pathogens with increased clinical relevance in immunocompromised patients. Because asymptomatic viral replication is frequently observed after kidney transplantation, only clinically significant infectious episodes were included in the present analysis. We differentiated seroconversion or asymptomatic viral replication from clinically significant infection. Clinically significant infection was defined by compatible symptoms, organ dysfunction, need for targeted antimicrobial treatment, and/or modification of immunosuppressive therapy. Infections requiring hospitalization, intensified monitoring, or additional diagnostic evaluation because of suspected graft involvement were also considered clinically significant when judged relevant in the overall clinical context. Isolated asymptomatic polymerase chain reaction (PCR) positivity without clinical findings, treatment modification, or documented impact during follow-up was categorized as asymptomatic infection/viremia and was not classified as an infection episode.

Viral infections, including CMV, EBV, and BKV, were diagnosed using PCR assays in blood samples. BKV infection was defined as clinically relevant viremia requiring modification of immunosuppressive therapy and/or additional therapeutic intervention [12,13]. BKV-associated nephropathy was recorded separately and required histological confirmation on kidney graft biopsy [12,13,14]. The diagnosis of BKV-associated nephropathy was based on compatible histopathological findings in conjunction with clinically significant viremia and graft-function deterioration.

CMV and EBV episodes were included only when DNAemia was accompanied by clinical findings, cytopenias, organ involvement, graft-function abnormalities, or therapeutic/immunosuppressive intervention [8,11]. Isolated low-level DNAemia requiring observation alone and showing no clinical significance during follow-up was not included as an opportunistic infection event.

Fungal infections were diagnosed based on compatible clinical findings supported by histopathological confirmation and/or molecular testing, including PCR from bronchoalveolar lavage samples when available. Only clinically significant infections requiring antifungal therapy were included.

West Nile virus (WNV) infection was included as a clinically significant infection occurring under chronic post-transplant immunosuppression, because neuroinvasive disease developed in the setting of long-term immunosuppressive treatment and required diagnostic evaluation and therapeutic intervention.

For the purposes of this study, infections were classified as early-onset if they occurred within the first 12 months after transplantation, including month 12, and late-onset if they occurred beyond 12 months. The 12-month threshold was selected pragmatically because the first post-transplant year is generally considered a distinct clinical period characterized by intensified immunosuppression, routine prophylactic strategies, and structured virological monitoring. Infections occurring beyond this period may reflect different patterns of exposure and susceptibility during long-term maintenance immunosuppression.

### 2.5. Outcomes

The primary outcome was the occurrence of clinically significant opportunistic infection following kidney transplantation. Secondary outcomes included infection type, timing of onset, management, clinical course, and impact on graft function, assessed relative to each patient’s baseline kidney function. Clinical outcomes were evaluated both at the patient level and according to individual infection episodes. Incidence was assessed at the patient level, while pathogen distribution and timing were described per infection episode. For patients with multiple infectious episodes, each event was analyzed independently with respect to timing, management and clinical outcome.

Graft dysfunction was defined as a sustained decrease in estimated glomerular filtration rate (eGFR) of 20% or more relative to each patient’s baseline value, documented by at least two consecutive measurements to exclude transient fluctuations. For the purposes of clinical assessment within this cohort, this threshold was used as a pragmatic indicator of clinically relevant graft impairment after exclusion of alternative causes of renal deterioration, including dehydration, hemodynamic instability, drug-related nephrotoxicity, or acute rejection. e-GFR was assessed mainly using the pediatric bedside Schwartz formula and, when available, cystatin C measurements. In cases of severe deterioration of graft function, GFR was further measured using DTPA renal scintigraphy. Kidney graft biopsy was performed when clinically indicated, particularly in cases of persistent graft dysfunction, suspected rejection, or possible BKV-associated nephropathy.

### 2.6. Statistical Analysis

Data were analyzed using descriptive statistical methods. Continuous variables are presented as mean (range) or median (range), as appropriate according to data distribution, whereas categorical variables are presented as frequencies and percentages. Because of the relatively small sample size, the limited number of infection episodes, and the heterogeneity in pathogen type and clinical presentation, the analysis was intentionally focused on descriptive characterization rather than formal hypothesis-testing statistical comparisons, in order to avoid overinterpretation of underpowered observations.

Incidence was evaluated at the patient level, while pathogen distribution, timing of infection, management strategies, and clinical outcomes were analyzed per infection episode. Longitudinal assessment focuses on infection-related changes in graft function, therapeutic interventions and infection resolution or persistence during follow-up. All statistical analyses were performed using IBM SPSS Statistics for Windows, version 28.0 (IBM Corp., Armonk, NY, USA).

### 2.7. Ethical Considerations

Ethical review and approval were waived by the institutional ethics committee due to the retrospective observational design of the study and the use of anonymized clinical data. The study was conducted in accordance with institutional policies and the principles of the Declaration of Helsinki. The requirement for informed consent was also waived. All data were handled in accordance with institutional confidentiality standards, and no patient-identifiable information was included in the analysis.

## 3. Results

### 3.1. Patient Characteristics

The study included 27 pediatric kidney transplant recipients. The mean age at transplantation was 10.2 years (range 3.5–18 years), and the mean duration of follow-up was 5.6 years (range 1–10 years). Most patients received grafts from deceased donors (85.2%), while living-donor transplantation was performed in 14.8% of cases. All patients were managed using standard immunosuppressive protocols.

The most common underlying cause of ESKD was congenital anomalies of the kidney and urinary tract (CAKUT), accounting for 66.7% of the cohort. Other underlying diagnoses included thrombotic microangiopathy (11.1%), infantile polycystic kidney disease (11.1%), focal segmental glomerulosclerosis (3.7%), and Bardet–Biedl syndrome (7.4%). Baseline demographic and clinical characteristics of the study population are summarized in Table 1.

### 3.2. Incidence of Opportunistic Infections

Clinically significant opportunistic infections were documented in 10 of 27 pediatric kidney transplant recipients (37.0%). One patient developed two distinct infectious episodes during follow-up, resulting in a total of 11 infection events. Incidence was assessed at the patient level, whereas pathogen distribution, timing, management and clinical outcomes were analyzed per infection episode. Overall, more than one-third of the cohort experienced at least one clinically significant opportunistic infection during long-term follow-up. Patients who developed opportunistic infections included both male and female recipients, were predominantly transplanted for CAKUT, and experienced infectious episodes across a broad range of ages and follow-up periods, reflecting the clinical heterogeneity of the affected subgroup.

### 3.3. Type of Infections

BKV was the most frequently identified pathogen, accounting for 4 of 11 infection episodes (36.4%). Fungal infections represented the second most frequent category (27.3%), followed by cytomegalovirus infection (18.2%). EBV and WNV infections were each recorded in one patient. Fungal infections included mucormycosis and *Pneumocystis jirovecii* pneumonia. The distribution of infectious agents is presented in Table 2. Viral pathogens predominated among infection episodes, with BKV representing the most frequent viral infection in this cohort.

### 3.4. Timing of Infections

**Figure 1:**
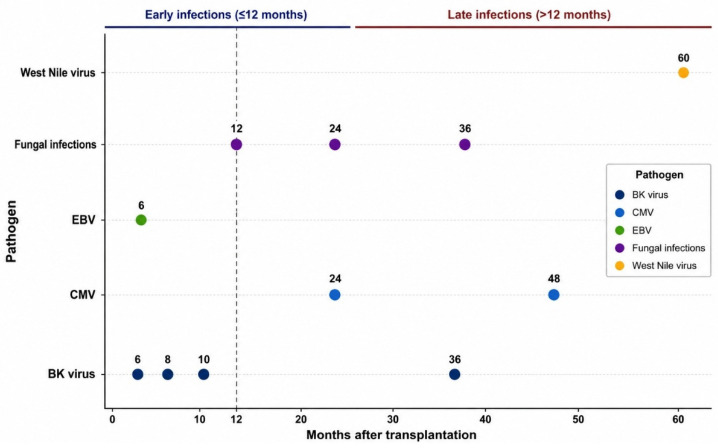
Timing of opportunistic infections after kidney transplantation, by pathogen type. Each point represents an individual infection event plotted according to time after transplantation (months). Colors correspond to pathogen type: BK virus (dark blue), cytomegalovirus (CMV) (light blue), Epstein–Barr virus (EBV) (green), fungal infections (purple) and West Nile virus (orange).

Infectious episodes were observed both within and beyond the first post-transplant year. Five events (45.5%) occurred within the first 12 months after transplantation, whereas 6 events (54.5%) were documented beyond 12 months (Figure 1). Early infections included BKV infection, EBV infection and one case of mucormycosis, whereas late infections included cytomegalovirus infection, fungal infections, BKV infection and WNV infection. The timing of infection episodes demonstrated that clinically significant opportunistic infections were not restricted to the early post-transplant period.

### 3.5. Diagnostic Approach and Clinical Course

All recorded infections were clinically significant and were confirmed by laboratory, histopathological, and/or molecular findings. Viral infections were identified using PCR-based assays, whereas BKV-associated nephropathy required histological confirmation on kidney graft biopsy. Fungal infections were diagnosed by histopathological examination and/or molecular testing.

Management strategies were individualized according to pathogen type, infection severity, and graft-function status. Infection-specific management approaches and clinical outcomes are summarized in Table 3.

Immunosuppressive therapy was reduced in all patients with BKV infection, while selected patients additionally received intravenous immunoglobulin and/or cidofovir. One of four patients with BKV infection developed biopsy-confirmed BKV-associated nephropathy associated with persistent graft dysfunction despite therapeutic intervention, whereas the remaining patients achieved virological remission or maintained stable graft function during follow-up.

Both CMV infections were associated with respiratory involvement and mild deterioration of kidney function. One late CMV episode, occurring four years after transplantation, presented pneumonia, transient cytopenias, and persistent viremia requiring adjunctive CMV-specific immunoglobulin. Both patients responded favorably to antiviral treatment without persistent graft dysfunction. EBV infection resolved following reduction of immunosuppression, intravenous immunoglobulin administration and close monitoring, without progression to post-transplant lymphoproliferative disease.

Fungal infections included one episode of *Pneumocystis jirovecii* pneumonia and two cases of mucormycosis. All patients received targeted antifungal treatment, while one patient with pulmonary mucormycosis additionally underwent surgical wedge resection. WNV infection followed a self-limited clinical course under supportive management and reduction of immunosuppressive therapy.

No infection-related mortality was observed during the study period. Preserved graft function was documented in most patients, whereas persistent graft dysfunction occurred only in the patient with biopsy-confirmed BKV-associated nephropathy. Detailed characteristics of individual infection episodes are summarized in Table 4.

## 4. Discussion

In this 10-year single-center cohort, clinically significant opportunistic infections were documented in 10 of 27 pediatric kidney transplant recipients. The episode-level review demonstrated substantial variability in pathogen type, timing, management and clinical outcome. Although the cohort was small, clinically significant infections were not restricted to the early post-transplant period, as more than half of all infectious episodes occurred beyond the first post-transplant year despite routine prophylaxis and virological surveillance [3,5,7].

Compared with previous pediatric kidney transplant studies that have primarily focused on individual viral pathogens, our study evaluated the broader spectrum of clinically significant opportunistic infections and their clinical consequences during long-term follow-up. An additional strength is the episode-based assessment of infection timing and outcomes, which allowed characterization of both early and late infectious complications beyond the first post-transplant year.

To place our findings in the context of the existing literature, Table 5 summarizes selected pediatric kidney transplant studies published during the last decade that evaluated post-transplant infectious complications. While most previous studies focused primarily on viral infections, particularly CMV, EBV, and BK virus, our study assessed the broader spectrum of clinically significant opportunistic infections and their clinical impact during long-term follow-up.

The timing of infection is clinically relevant. The first year after transplantation is generally considered the period of highest infectious risk because of intensified immunosuppression and early post-operative vulnerability [3,5]. However, in our cohort, late infections included CMV, fungal infections, BKV and WNV. These findings support maintaining opportunistic infections in the differential diagnosis during long-term follow-up, particularly in patients presenting with new symptoms or otherwise unexplained graft dysfunction [8,9].

BKV was the most frequent pathogen, accounting for 4 infection episodes. Clinical presentation and outcomes varied substantially among affected patients, ranging from transient viremia with virological remission after reduction of immunosuppression to biopsy-confirmed BKV-associated nephropathy associated with persistent graft dysfunction. Reported risk factors for BKV infection in pediatric kidney transplant recipients include younger recipient age, male sex, intensified immunosuppression, deceased donor transplantation, acute rejection and urological abnormalities such as obstructive uropathy or CAKUT. In our cohort, these observations were partly reflected, as BKV infection was mainly observed in children with CAKUT and during the first post-transplant year, while the only case of biopsy-confirmed BKV-associated nephropathy occurred in the youngest affected patient [15,17]. This pattern is consistent with previous reports showing that BKV infection may range from controllable viremia to nephropathy with potential impact on graft function [12,13,14,15,16,17].

These observations underscore the variable clinical course of BKV infection and support individualized interpretation of BK viremia according to viral kinetics, graft function, histopathological findings, and the overall immunological context [12,13]. In the present cohort, BKV accounted for the only case of sustained infection-related graft dysfunction, further supporting its continued relevance in pediatric post-transplant surveillance.

Both CMV episodes occurred late, at 24 and 48 months after transplantation. In both cases, CMV infection was associated with respiratory tract involvement and mild deterioration of kidney function rather than isolated PCR positivity. Treatment with antiviral therapy and CMV-specific immunoglobulin was followed by clinical recovery without persistent graft dysfunction. These observations demonstrate that clinically significant CMV disease may occur despite prolonged prophylaxis. Continued clinical vigilance is therefore warranted in patients presenting with respiratory symptoms or unexplained deterioration of graft function [7,11,18].

EBV was recorded in one patient during the early post-transplant period. The episode occurred in a 16-year-old patient with recurrent focal segmental glomerulosclerosis who required intensified immunosuppression because of early recurrence of the primary disease. EBV DNAemia was accompanied by transient neutropenia and thrombocytopenia, while graft function remained stable and symptoms were minimal. Immunosuppression was reduced, intravenous immunoglobulin was administered, and viral clearance was achieved within one month. No post-transplant lymphoproliferative disease (PTLD) was observed. Despite the favorable clinical course observed in our patient, EBV replication remains clinically important in pediatric transplant recipients because of its established association with PTLD, particularly under intensified immunosuppression [6,7,8].

Fungal infections included two cases of mucormycosis and one case of *Pneumocystis jirovecii* pneumonia. Although few, these infections were clinically important because invasive fungal infections in solid organ transplant recipients may progress rapidly and may present with non-specific findings [19,20,21]. Diagnosis was based on imaging together with histopathological and/or molecular confirmation, while treatment was guided by infection site and severity. Two of the three fungal infections occurred beyond the first post-transplant year, indicating that fungal disease should also be considered during later follow-up. The favorable outcomes may have been facilitated by early clinical suspicion, prompt diagnostic evaluation, and timely initiation of targeted antifungal therapy.

WNV infection was identified in one patient 5 years after transplantation. The patient presented with fever and headache followed by acute neurological symptoms and was diagnosed with encephalitis after exclusion of other infectious causes and detection of serum anti-WNV IgM antibodies. Although WNV is not traditionally classified among latent opportunistic viral infections, neuroinvasive disease developed in the setting of chronic immunosuppression and required extensive diagnostic evaluation. The patient improved after supportive management and reduction of immunosuppression. This case supports consideration of WNV infection in transplant recipients presenting with fever or neurological manifestations in regions where the virus circulates seasonally [22,23].

Overall outcomes were favorable. Most infections were resolved after targeted management, graft function was preserved in most patients, and no infection-related mortality was observed. The main exception was the patient with BKV-associated nephropathy, who developed persistent graft dysfunction. Accordingly, even within a cohort characterized by generally favorable outcomes, selected opportunistic infections may still exert a sustained negative effect on graft function.

Several limitations should be acknowledged. The study was retrospective, single-center, and included a relatively small number of patients, limiting generalizability and precluding formal analysis of infection risk factors. Management strategies were not entirely uniform, reflecting individualized clinical decision-making and evolving transplant practices during the study period. In addition, only clinically significant infections were included; therefore, low-level or asymptomatic viral DNAemia was not analyzed. Nevertheless, the present cohort reflects real-world clinical practice in a highly specialized pediatric kidney transplant population. Furthermore, detailed pediatric data regarding opportunistic infections, particularly beyond the first post-transplant year, remain relatively limited in the literature.

Despite these limitations, the present study provides a clinically focused characterization of opportunistic infections in pediatric kidney transplant recipients followed over an extended period. Clinically significant infections occurred both early and late after transplantation, with more than half of all infectious episodes developing beyond the first post-transplant year. BKV represented the predominant pathogen and accounted for the only case of persistent infection-related graft dysfunction. Taken together, these findings support the need for continued infectious surveillance and sustained clinical vigilance throughout long-term pediatric post-transplant follow-up.

## 5. Conclusions

In this 10-year single-center cohort, clinically significant opportunistic infections affected more than one-third of pediatric kidney transplant recipients and occurred both during the early and late post-transplant periods. More than half of all infectious episodes developed beyond the first post-transplant year despite routine prophylaxis and virological surveillance. BKV represented the predominant pathogen and accounted for the only case of persistent graft dysfunction due to biopsy-confirmed BKV-associated nephropathy. Although overall outcomes were favorable and no infection-related mortality was observed, these findings underscore the importance of sustained infectious surveillance and continued clinical vigilance throughout long-term pediatric post-transplant follow-up.

## Figures and Tables

**Table 1:** Baseline characteristics of pediatric kidney transplant recipients.

Characteristic	Value
Total patients	27
Age at transplantation, years, mean (range)	10.2 (3.5–18)
Follow-up duration, years, mean (range)	5.6 (1–10)
**Sex**	
Male	16 (59.3%)
Female	11 (40.7%)
**Donor type**	
Living donor	4 (14.8%)
Deceased donor	23 (85.2%)
**Underlying kidney disease**	
CAKUT	18 (66.7%)
Thrombotic microangiopathy	3 (11.1%)
Infantile polycystic kidney disease	3 (11.1%)
Focal segmental glomerulosclerosis	1 (3.7%)
Bardet–Biedl syndrome	2 (7.4%)

Values are presented as n (%) unless otherwise indicated. Percentages were calculated using the total cohort of 27 patients. CAKUT, congenital anomalies of the kidney and urinary tract.

**Table 2:** Distribution of opportunistic infections by pathogen type.

Infection Type	Infection Events, n (%)
BK virus	4 (36.4)
Fungal infections	3 (27.3)
Cytomegalovirus	2 (18.2)
Epstein–Barr virus	1 (9.1)
West Nile virus	1 (9.1)
**Total**	11 (100)

Percentages were calculated per infection episode. Eleven infection episodes occurred in 10 patients; one patient experienced two distinct infections.

**Table 3:** Management and outcomes of opportunistic infections in pediatric kidney transplant recipients.

Infection Type	Patients, n	Management	Outcome
BK virus	4	Reduction of immunosuppression; IVIG in selected cases; cidofovir in 1 patient	BK virus-associated nephropathy in 1; remission in 2; stable graft function in 1
Cytomegalovirus	2	Antiviral therapy; CMV-specific immunoglobulin	Full recovery
Epstein–Barr virus	1	Reduction of immunosuppression; IVIG	Viral clearance; no PTLD
Fungal infections	3	Antifungal therapy +/− surgical intervention	Infection resolution
West Nile virus	1	Supportive care; reduction of immunosuppression	Full recovery

Outcomes are summarized at the patient level within each infection category. BK virus-associated nephropathy was observed in one patient and was associated with graft dysfunction. CMV, cytomegalovirus; IVIG, intravenous immunoglobulin; PTLD, post-transplant lymphoproliferative disorder.

**Table 4:** Clinical characteristics, management, and outcomes of individual opportunistic infection episodes.

Episode	Age at Infection, Years	Underlying Disease	Pathogen	Time After Transplantation, Months	Timing	Diagnostic Method	Management	Outcome
1	4	CAKUT	BK virus	6	Early	Blood PCR; biopsy confirmation of BKVN	Reduction of immunosuppression, IVIG, cidofovir	BKVN with graft dysfunction
2	7	CAKUT	BK virus	<12	Early	Blood PCR	Reduction of immunosuppression, IVIG	Virological remission
3	12	TMA	BK virus	<12	Early	Blood PCR	Reduction of immunosuppression, IVIG	Virological remission
4	14	CAKUT	BK virus	36	Late	Blood PCR	Reduction of immunosuppression	Stable graft function
5	12	CAKUT	CMV	24	Late	Blood PCR	Valganciclovir followed by intravenous ganciclovir, CMV-specific immunoglobulin	Recovery
6	15	BBS	CMV	48	Late	Blood PCR; imaging assessment	Intravenous ganciclovir, CMV-specific immunoglobulin	Resolution
7	16	FSGS	EBV	12	Early	Blood PCR	IVIG, reduction of immunosuppression	Viral clearance; no PTLD
8	16	CAKUT	Mucormycosis	12	Early	PCR on biopsy specimen	Amphotericin B followed by posaconazole	Recovery
9	16	CAKUT	Mucormycosis	36	Late	Histopathology and PCR	Surgical intervention and antifungal therapy	Recovery
10	15	CAKUT	*Pneumocystis jirovecii* pneumonia	24	Late	PCR, β-D-glucan	Trimethoprim-sulfamethoxazole	Recovery
11	18	TMA	West Nile virus	60	Late	IgM serology	Supportive care, reduction of immunosuppression	Recovery

Eleven infection episodes occurred in 10 patients; one patient experienced two distinct infections. Age refers to age at the time of infection. Early infections were defined as those occurring within 12 months after transplantation, including month 12; late infections were defined as those occurring beyond 12 months. BBS, Bardet–Biedl syndrome; BKVN, BK virus-associated nephropathy; CAKUT, congenital anomalies of the kidney and urinary tract; CMV, cytomegalovirus; EBV, Epstein–Barr virus; FSGS, focal segmental glomerulosclerosis; IVIG, intravenous immunoglobulin; PCR, polymerase chain reaction; PTLD, post-transplant lymphoproliferative disease; TMA, thrombotic microangiopathy.

**Table 5:** Selected pediatric kidney transplant studies evaluating post-transplant infectious complications published during the last decade.

Study	Year	Design	Patients (n)	Infection Focus	Key Findings
[7]	2019	Retrospective single-center cohort	92	CMV, EBV, and BKV infections	Viral infections occurred in 84% of recipients, with EBV being the most frequent pathogen. Most episodes were asymptomatic, while CMV disease was uncommon despite universal valganciclovir prophylaxis
[15]	2021	Retrospective single-center cohort	106	BK virus infection and BK virus nephropathy	BK viremia occurred in 30.2% and BKVN in 6.6% of recipients. Younger age at transplantation and higher immunosuppressive burden were associated with infection
[6]	2022	Retrospective single-center cohort	67	CMV, EBV, and BKV infections	Viral infections occurred in 35.2% of recipients. Younger age and thymoglobulin induction were associated with infection, while long-term graft outcomes remained generally favorable
[16]	2024	Retrospective single-center cohort	79	Viral infections (CMV, BKV, VZV, parvovirus B19, COVID-19)	Viral infections occurred in 23% of recipients. BKV was the most frequent pathogen, and infected patients showed lower long-term graft function and higher graft loss rates
Present study	2026	Retrospective single-center cohort	27	Clinically significant opportunistic infections (BKV, CMV, EBV, fungal infections, and WNV)	Clinically significant opportunistic infections occurred in 37.0% of recipients. More than half of all infectious episodes developed beyond the first post-transplant year. BKV accounted for the only case of persistent infection-related graft dysfunction, while overall outcomes were favorable and no infection-related mortality was observed

Selected studies evaluating post-transplant infectious complications in pediatric kidney transplant recipients published during the last decade. BKV, BK virus; BKVN, BK virus-associated nephropathy; CMV, cytomegalovirus; COVID-19, coronavirus disease 2019; EBV, Epstein–Barr virus; VZV, varicella-zoster virus; WNV, West Nile virus.

## Data Availability

The data presented in this study are available on request from the corresponding author. The data are not publicly available due to privacy and ethical restrictions involving pediatric transplant recipients.

## Abbreviations

The following abbreviations are used in this manuscript:

ESKD	End-Stage Kidney Disease
CMV	Cytomegalovirus
EBV	Epstein–Barr Virus
BKV	BK Virus
WNV	West Nile Virus
PCR	Polymerase Chain Reaction
eGFR	estimated Glomerular Filtration Rate
CAKUT	Congenital Anomalies of the Kidney and Urinary Tract
PTLD	Post-Transplant Lymphoproliferative Disease

## References

[1] Winterberg P.D., Garro R. (2019). Long-term outcomes of kidney transplantation in children. Pediatr. Clin. N. Am..

[2] Verghese P.S. (2017). Pediatric kidney transplantation: A historical review. Pediatr. Res..

[3] Fishman J.A. (2017). Infection in organ transplantation. Am. J. Transplant..

[4] Danziger-Isakov L., Kumar D., AST Infectious Diseases Community of Practice (2019). Vaccination of solid organ transplant candidates and recipients: Guidelines from the American Society of Transplantation Infectious Diseases Community of Practice. Clin. Transplant..

[5] Fishman J.A. (2007). Infection in solid-organ transplant recipients. N. Engl. J. Med..

[6] Levi S., Davidovits M., Alfandari H., Dagan A., Borovitz Y., Bilavsky E., Landau D., Haskin O. (2022). EBV, CMV, and BK viral infections in pediatric kidney transplantation: Frequency, risk factors, treatment, and outcomes. Pediatr. Transplant..

[7] Paulsen G., Cumagun P., Mixon E., Fowler K., Feig D., Shimamura M. (2019). Cytomegalovirus and Epstein-Barr virus infections among pediatric kidney transplant recipients: Incidence and timing of infections. Pediatr. Transplant..

[8] Allen U.D., Preiksaitis J.K., AST Infectious Diseases Community of Practice (2019). Post-transplant lymphoproliferative disorders, Epstein-Barr virus infection, and disease in solid organ transplantation: Guidelines from the American Society of Transplantation Infectious Diseases Community of Practice. Clin. Transplant..

[9] Kasiske B.L., Zeier M.G., Craig J.C., Kreis H.A., Ekberg H., Green M. (2009). Kidney Disease: Improving Global Outcomes (KDIGO) Transplant Work Group. KDIGO clinical practice guideline for the care of kidney transplant recipients. Am. J. Transplant..

[10] Fishman J.A., Gans H., AST Infectious Diseases Community of Practice (2019). *Pneumocystis jiroveci* in solid organ transplantation: Guidelines from the American Society of Transplantation Infectious Diseases Community of Practice. Clin. Transplant..

[11] Kotton C.N., Kumar D., Caliendo A.M., Huprikar S., Chou S., Danziger-Isakov L., Humar A., The Transplantation Society International CMV Consensus Group (2018). The third international consensus guidelines on the management of cytomegalovirus in solid-organ transplantation. Transplantation.

[12] Hirsch H.H., Randhawa P.S., AST Infectious Diseases Community of Practice (2019). BK polyomavirus in solid organ transplantation: Guidelines from the American Society of Transplantation Infectious Diseases Community of Practice. Clin. Transplant..

[13] Kotton C.N., Kamar N., Wojciechowski D., Eder M., Hopfer H., Randhawa P., Sester M., Comoli P., Silva H.T., Knoll G. (2024). The second international consensus guidelines on the management of BK polyomavirus in kidney transplantation. Transplantation.

[14] Hirsch H.H., Brennan D.C., Drachenberg C.B., Ginevri F., Gordon J., Limaye A.P., Mihatsch M.J., Nickeleit V., Ramos E., Randhawa P. (2005). Polyomavirus-associated nephropathy in renal transplantation: Interdisciplinary analyses and recommendations. Transplantation.

[15] McCaffrey J., Bhute V.J., Shenoy M. (2021). BK virus infection and outcome following kidney transplantation in childhood. Sci. Rep..

[16] Parmaksiz G., Avci B., Noyan A., Baştürk B., Çalişkan K., Baskin E., Haberal M. (2024). Viral Infections in Pediatric Kidney Transplant Recipients: Effects on Graft Function, Risk Factors, and Patient Outcomes. Exp. Clin. Transplant..

[17] Chiodini B., Guillaume-Gentil P., Vanhomwegen C., Hennaut E., Lolin K., Tram N., Le Moine A., Ismaili K. (2024). BK polyomavirus in pediatric renal transplantation-what do we know so far?. Biomedicines.

[18] Kotton C.N., Kumar D., Manuel O., Chou S., Hayden R.T., Danziger-Isakov L., Asberg A., Tedesco-Silva H., Humar A., on behalf of The Transplantation Society International CMV Consensus Group (2025). The fourth international consensus guidelines on the management of cytomegalovirus in solid organ transplantation. Transplantation.

[19] Pappas P.G., Alexander B.D., Andes D.R., Hadley S., Kauffman C.A., Freifeld A., Anaissie E.J., Brumble L.M., Herwaldt L., Ito J. (2010). Invasive fungal infections among organ transplant recipients: Results of the Transplant-Associated Infection Surveillance Network. Clin. Infect. Dis..

[20] Palomba E., Colaneri M., Azzarà C., Fava M., Maccaro A., Renisi G., Viero G., Kaur H., Chakrabarti A., Gori A. (2024). Epidemiology, clinical manifestations, and outcome of mucormycosis in solid organ transplant recipients: A systematic review of reported cases. Open Forum Infect. Dis..

[21] Dotis J., Printza N., Stabouli S., Karava V., Gkogka C., Vyzantiadis T.A., Roilides E., Papachristou F. (2019). Disseminated mucormycosis in an adolescent kidney transplant recipient. Kidney Int..

[22] Anesi J.A., Silveira F.P., AST Infectious Diseases Community of Practice (2019). Arenaviruses and West Nile virus in solid organ transplant recipients: Guidelines from the American Society of Transplantation Infectious Diseases Community of Practice. Clin. Transplant..

[23] Kasule S.N., Razonable R.R., Blair J.E. (2023). Neuroinvasive West Nile virus infection in solid organ transplant recipients. Transpl. Infect. Dis..

